# Evaluation of the Toxic Potential of Graphene Copper Nanocomposite (GCNC) in the Third Instar Larvae of Transgenic *Drosophila melanogaster (hsp70-lacZ)Bg^9^*

**DOI:** 10.1371/journal.pone.0080944

**Published:** 2013-12-05

**Authors:** Yasir Hasan Siddique, Ambreen Fatima, Smita Jyoti, Falaq Naz, Rahul, Wasi Khan, Braj Raj Singh, Alim Hussain Naqvi

**Affiliations:** 1 Drosophila Transgenic Laboratory, Section of Genetics, Department of Zoology, Faculty of Life Sciences, Aligarh Muslim University, Aligarh, Uttar Pradesh, India; 2 Centre of Excellence in Materials Sciences (Nano materials), Department of Applied Physics, Z.H. College of Engineering & Technology, Aligarh Muslim University, Aligarh, Uttar Pradesh, India; Alexander Fleming Biomedical Sciences Research Center, Greece

## Abstract

Graphene, a two-dimensional carbon sheet with single-atom thickness, have attracted the scientific world for its potential applications in various field including the biomedical areas. In the present study the graphene copper nanocomposite (GCNC) was synthesized, characterized and evaluated for its toxic potential on third instar larvae of transgenic *Drosophila melanogaster (hsp70-lacZ)Bg^9^*. The synthesized GCNC was analyzed by X-ray diffraction (XRD), scanning/transmission electron microscopy (SEM/TEM), atomic force microscopy (AFM), and fourier transform infrared spectroscopy (FTIR). The GCNC in 0.1% DMSO was sonicated for 10 min and the final concentration of 0.033, 0.099, 0.199 and 3.996 µg/µl of diet were established. The third instar larvae were allowed to feed on it separately for 24 and 48 hrs. The *hsp70* expression was measured by O-nitrophenyl-β-D-galactopyranoside assay, tissue damage by trypan blue exclusion test and β-galactosidase activity was monitored by *in situ* histochemical β-galactosidase staining. Oxidative stress was monitored by performing lipid peroxidation assay and total protein estimation. Ethidium bromide/acridine orange staining was performed on midgut cells for apoptotic index and the comet assay was performed for the DNA damage. The results of the present study showed that the exposure of 0.199 and 3.996 µg/µl of GCNC were toxic for 24 hr of exposure and for 48 hr of exposure: 0.099, 0.199 and 3.996 µg/µl of GCNC was toxic. The dose of 0.033 µg/µl of GCNC showed no toxic effects on its exposure to the third instar larvae for 24 hr as well as 48 hrs. This dose can be considered as No Observed Adverse Effect Level (NOAEL).

## Introduction

The unique physiochemical properties of graphene and its derivatives have attracted great research interest for their potential applications in electronics, energy, materials and biomedical areas [1]. As compared to carbon nanotubes (CNTs) graphene has no impurities thus providing an advantage for the construction of reliable sensors, as well as energy storage devices [2], [3]. Many studies have shown that nanomaterials and their derivative may have negative effects on health [4]. The most widely used and studied graphene derivatives is graphene oxide (GO), commonly used to produce the graphene based nanocomposites [5]. The morphology of the graphene is quite distinct from carbon nanotubes. The length of the carbon nanotubes can influence its toxicity but graphene sheets and GO do not have length [6]. Single walled carbon nano tubes (SWNTs) administered orally at 1000 mg/kg body weight in mice did not show any toxic or behavioural changes. However, the intra peritoneal administration of SWNTs coalese inside the body and induced granuloma formation [7]. Multiwall carbon nano tubes (CNT) administered intratracheally to sprague-Dawley rats showed inflammatory and fibrotic reactions [8]. GO can form conjugates with various systems such as polymers, biomolecules, DNA, protein, quantum dots, and others making GO usable for various biological and medical applications [9]. The graphene nanocomposite containing poly N-Vinylcarbazole and graphene solutions in the form of thin film was evaluated for its compatibility on NIH 3T3 cells using MTS cell proliferation assay and after 24 hr of exposure about 80% cell survival was reported [10]. The full implementation of such nonmaterials in a large range of biological applications and processes needs an insight into the interaction between graphene composites and various biological systems both *in vitro* and *in vivo* [11]. In this context, the graphene copper nanocomposite (GCNC) was synthesized, characterized and evaluated for its toxic potential at various doses on the third instar larvae of transgenic *Drosophila melanogaster (hsp70-lacZ)Bg^9^*.

## Materials and Methods

### Synthesis of Graphene-Cu_2_O Nanocomposite (GCNC)

Graphite oxide (GO) was prepared according to the method described by Hummers and Offeman [12] from fine graphite powder. Briefly, 2 g of graphite powder and 1.5 g of NaNO_3_ were placed in a beaker. Then 100 ml of H_2_SO_4_ was added while stirring in an ice-water bath, and 25 g of KMnO_4_ was slowly added for 1 hr. Stirring was continued for 1 hr in the ice-water bath, and the mixture was again stirred at room temperature until it became pasty brownish. The pasty brownish mixture was then diluted with slowly addition of 200 ml water. The reaction temperature was rapidly increased to 90°C, and the color was changed to brown color after 1 hr. Finally, 10 ml of 30% aqueous solution of H_2_O_2_ was added to complete the oxidation. The impurities were removed from the graphene oxide (GO) by using 3% HCl aqueous solution by the repeated cycle of washing. The obtained GO was dried and stored for further use. The synthesis of the graphene-Cu_2_O (GCNC) nanocomposite involved two steps. In the first step, 100 mg of copper acetate and 200 mg of GO were dispersed into 200 ml of absolute ethanol and sonicated for 30 min. The resultant mixture was stirred for 1 hr, and then harvested by the centrifugation at 5000 rpm for 5 min and washed with 80% ethanol. The obtained sample was vacuum dried at 70°C for 10 hr and 100 mg of this dried sample was mixed with 100 ml of ethylene glycol under sonication in a 250 ml culture flask for 10 min. The resulting mixture was then heated to 140°C under vigorous magnetic stirring for 3 hr. The synthesized GCNC suspension was centrifuged washed by 80% ethanol three times to remove the remaining ethylene glycol, soluble by products, and dried in a vacuum oven at 60°C for 6 hr. The product was designated as GCNC and stored for further use.

### Characterization of GCNC

The synthesis of GCNC in solution was monitored by measuring the absorbance (A) using UV-Vis spectrophotometer (Perkin Elmer Life and Analytical Sciences, CT, USA) in the wavelength range of 200 to 800 nm. The vacuum dried GCNC powder was stored in amber color vials at room temperature under dry and dark condition. The X-ray diffraction (XRD) patterns of powdered sample was recorded on MiniFlex™ II benchtop XRD system (Rigaku Corporation, Tokyo, Japan) operating at 30 kV.

The average crystallite size (*d*) of Cu_2_O NPs was calculated following the Debye-Scherrer formula:
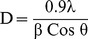



Where *k = *0.9 is the shape factor, λ is the X-ray wavelength of Cu Kα radiation (1.54 Å), θ is the Bragg diffraction angle, and β is the full width at half maximum height (FWHM) of the (101) plane diffraction peak. The microstructure and morphology analysis of sample was done using a JEOL transmission electron microscope (JEM-2010) and scanning electron microscope (JSM-6510LV) equipped with an energy dispersive spectrometer (EDS). For the morphological analysis transmission electron microscopy (TEM) of ethanol solution of GCNC was carried out on JEOL 100/120 kV transmission electron microscope (JEOL, Tokyo, Japan) with an accelerating voltage of 80 kV. For TEM analysis, a drop of GCNC was placed on the carbon coated copper grid and air dried under dark. The thin film of the GCNC was prepared on the borosilicate glass slide for the analysis of surface morphology. The prepared thin film was analyzed on the atomic force microscope (AFM; Innova SPM, Veeco) in tapping mode. The commercial etched silicon tips as scanning probes with typical resonance frequency of 300 Hz (RTESP, Veeco) was used. The microscope was placed on a pneumatic anti-vibration desk, under a damping cover and analysis was performed using the SPM Lab software. The electron and AFM images were obtained and converted into an enhanced meta file format. For the FTIR spectroscopic measurements GCNC powder was mixed with spectroscopic grade potassium bromide (KBr) in the ratio of 1∶100 and spectra recorded in the range of 400–4000 wave number (cm^−1^), on Perkin Elmer FTIR Spectrum BX (PerkinElmer Life and Analytical Sciences, CT, USA) in the diffuse reflectance mode at a resolution of 4 cm/In KBr pellets.

### Fly Strain

A transgenic *Drosophila melanogaster* line that expresses bacterial β-galactosidase as a response to stress was used in the present study [13]. In this strain of flies, the transformation vector is inserted with a P-element i.e. the line contains wild type *hsp70* sequence up to lac Z fusion point. The flies and larvae were cultured on standard *Drosophila* food containing agar, corn meal, sugar and yeast at 24±1°C [14].

### Experimental Design

GCNC in 0.1% DMSO was sonicated for 10 min and the final concentration 0.033, 0.099, 0.199 and 3.996 µg/µl of diet were established. The larvae were allowed to feed on it separately for 24 and 48 hrs. Untreated and negative controls (0.1% DMSO) were also run simultaneously. Graphene oxide nanoparticle (GONP) and Cuprous oxide nanoparticle (CONP) at 3.996 µg/ml of diet were also run as supplementary controls.

### Soluble O-nitrophenyl-β-D-galactopyranoside (ONPG) Assay

The expression of *hsp70* provides a measurement of cytotoxicity [15], [16]. The method described by Nazir et al [14] was used in this study. After washing in phosphate buffer, larvae were placed in a microcentrifuge tube (20 larvae/tubes, five replicates/groups), permeabilized for 10 min by acetone, and incubated overnight at 37°C in 600 µl of ONPG staining buffer. Following incubation, the reaction was stopped by adding 300 µl of Na_2_CO_3_. The extent of the reaction was quantified by measuring absorbance at 420 nm.

### Trypan Blue Exclusion Test

The extent of tissue damage in larvae caused by the exposure to different concentrations of GCNC was assayed by a dye exclusion test [14], [17]. Briefly, the internal tissues of larvae were explanted in a drop of Pole’s salt solution (PSS), washed in phosphate buffer saline (PBS), stained in trypan blue (0.2 mg/ml in PBS) for 30 min, washed thoroughly in PBS, and scored immediately for dark blue staining. About 50 larvae per treatment (10 larvae/dose; 5 replicates/group) were scored for the trypan blue staining on an average composite index per larvae: no color = 0; any blue = 1; darkly stained = 2; large patches of darkly stained cells = 3; or complete staining of most cells in the tissue = 4 [17].

### In situ Histochemical β-galactosidase Activity

The larvae (10 larvae/treatment; 5 replicates/group) were dissected out in PSS and X-gal staining was performed using the method as described by described by Chowdhuri et al. [15]. The tissue explants were fixed in 2.5% glutaraldehyde, washed in 50 mM sodium phosphate buffer (pH 8.0), and stained overnight in X-gal staining solution at 37°C in dark.

### Preparation of Larval Homogenate for Lipid Peroxidation Assay and Total Protein Content

The larvae (10 larvae/experiment; 5 replicates/group) were homogenized in 1 ml of cold homogenizing buffer (0.1 M Phosphate buffer containing 0.15 M KCl; pH 7.4). The supernatant after centrifugation at 9000 g was used for estimating lipid peroxidation and total protein content.

### Lipid Peroxidation Assay

Lipid peroxidation assay was performed as described earlier using 1,1,3,3-tetramethoxy propane as a standard [18]. Reagent 1 (R1) was prepared by dissolving 0.064 g of 1-methyl-2-phenylindole in 30 ml of acetonitrile to which 10 ml of methanol was added to bring the volume of 40 ml. The preparation of 37% HCl served as the reagent R2. About 100 µl of the supernatant, 650 µl of R1, and 150 µl of R2 was taken in the microcentrifuge tubes and vortexed. The tubes were incubated at 45°C for 45 mins. The tubes were then cooled in melting ice and readings were noted at 586 nm.

### Protein Estimation

Estimation of protein levels in all the treated as well as control groups was done according to the method of Bradford [19] using bovine serum albumin (BSA) as a standard.

### Assay to Detect Apoptosis

The apoptotic cells were analyzed by staining with an ethidium bromide (EB) and acridine orange (AO) staining. The midgut of the larvae was explanted in PSS. The PSS was replaced by 300 µl of collagenase (0.5 mg/ml) and kept for 15 min at 25°C. The collagenase was removed and the pellet was washed three times by PBS with gentle shaking [20]. Finally the pellet was suspended in 80 µl of PBS. About 25 µl of cell suspension was mixed with 2 µl of EB/AO dye. The staining dye was prepared by dissolving 100 µg/ml AO and 100 µg/ml EB in PBS. About 100 cells were scored per treatment (5 replicates/group) for estimating the apoptotic index and expressed in percent [21].

### Analysis of DNA Damage by Comet Assay

The comet assay was performed according to Mukhopadhyay et al. [20]. The midgut from 20 larvae per treatment (3 replicates/group) was explanted in PSS. PSS in microcentrifuge tube was replaced by 300 µl of collagenase (0.5 mg/ml in PBS, pH 7.4) and kept for 15 min at 25°C. The cell suspension was prepared by washing three times in PBS and finally the cells were suspended in 80 µl of PBS. The cell viability was checked by performing trypan blue assay before beginning the experiment [22]. About 75 µl of cell suspension was mixed with 80 µl of 1.5% low melting agarose and layered on top of the precoated slides with 1% normal melting point agarose. The slides were then immersed in freshly prepared chilled lysing solution (2.5 M NaCl, 100 mM EDTA, 10 mM Tris pH 10.0 and 1% Triton X-100, pH 10) for 2 h at 4°C. The slides were then transferred to the chilled electrophoresis solution (1 mM Na_2_ EDTA and 300 mM NaOH, pH >13). The slides were left in this solution for 10 min to allow DNA unwinding. Electrophoresis was conducted for 15 min at 0.7 V/cm and 300 mA at 4°C. Following electrophoresis, the slides were washed by neutralizing buffer (0.4 M Tris buffer) three times. Slides were then stained with ethidium bromide (20 µg/ml; 75 µl/slides) for 10 min in dark. The slides were then dipped in chilled distilled water to remove the excess of stain and subsequently cover slips were placed over them. Each experiment was performed in triplicate and the slides were prepared in duplicate. Twenty five cells per slide were randomly captured at a constant depth of the gel, and mean tail length (a.u) was calculated to measure DNA damage by using comet score 1.5 software (Comet Score™ v1.5 Software, TriTek Corporation, Sumerduck).

### Statistical Analysis

Student’s “t”-test and regression analysis were performed by using commercial software Statistica from Stat- Soft Inc.

## Results and Discussion

**Figure 1:**
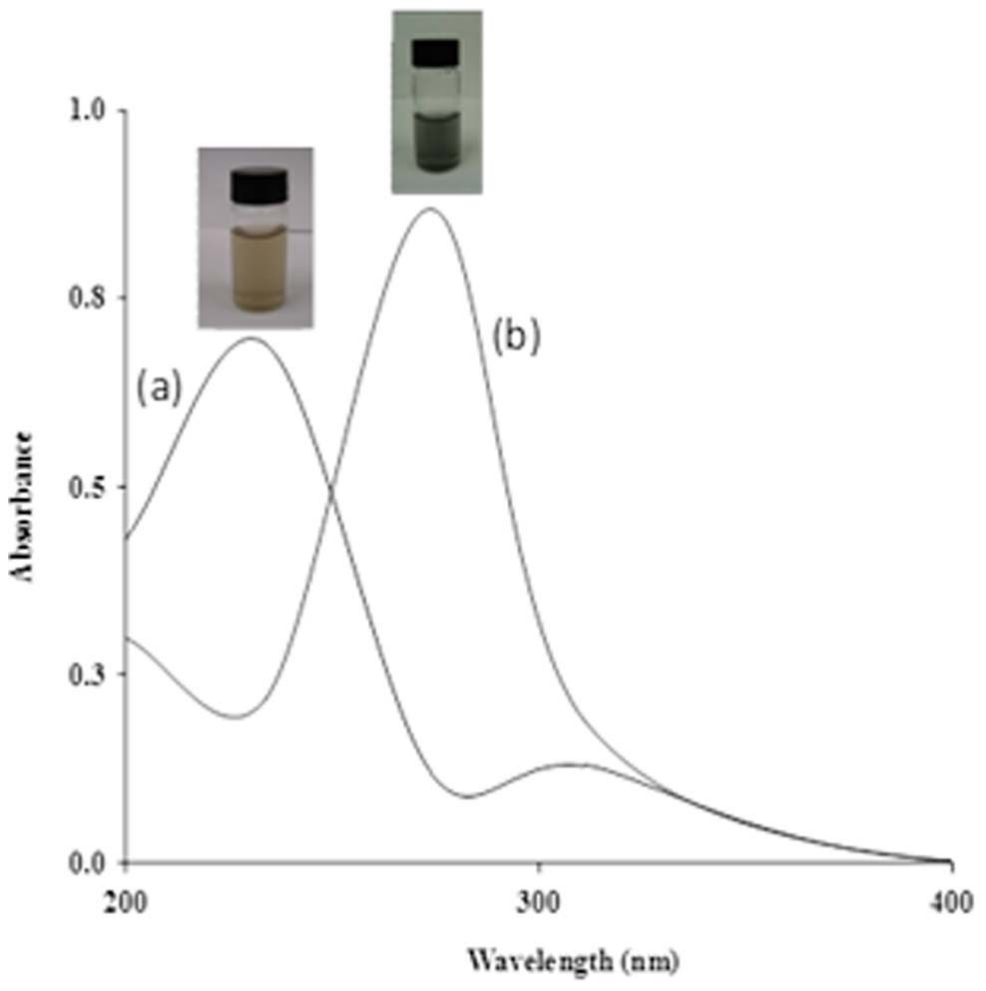
UV-Vis spectra for GO (a) and graphene-copper nanocomposite (b).

**Figure 2:**
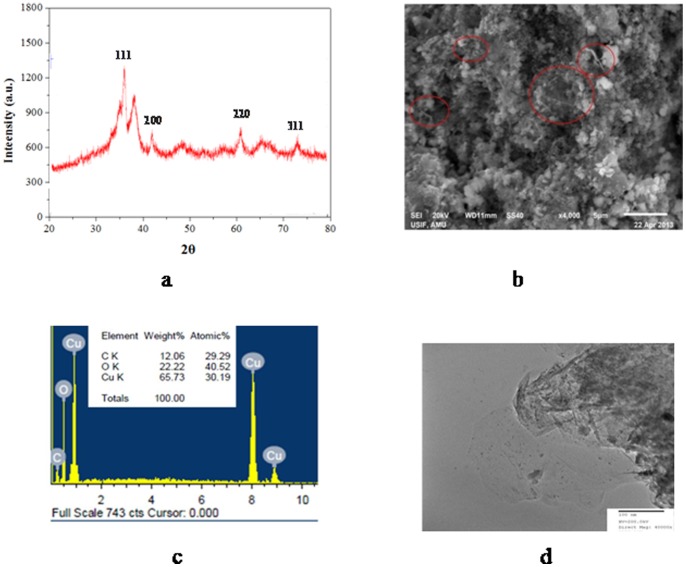
Figure 2a. XRD pattern of GCNC. Figure 2b. Scanning electron micrograph of GCNC. Figure 2c. Energy disperse spectrum of GCNC. Figure 2d. Transmission electron micrograph of the GCNC.

**Figure 3:**
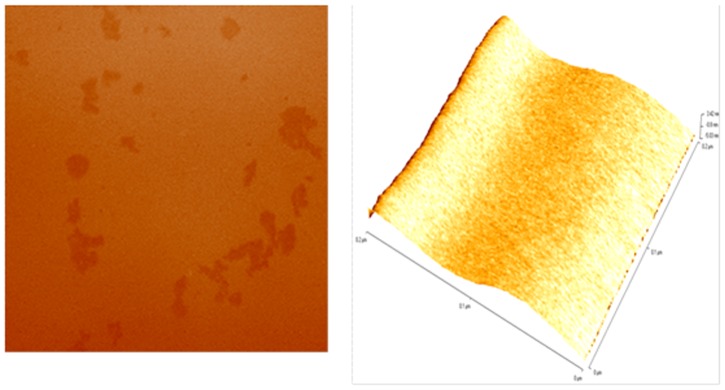
Atomic micrograph of the GCNC.

**Figure 4:**
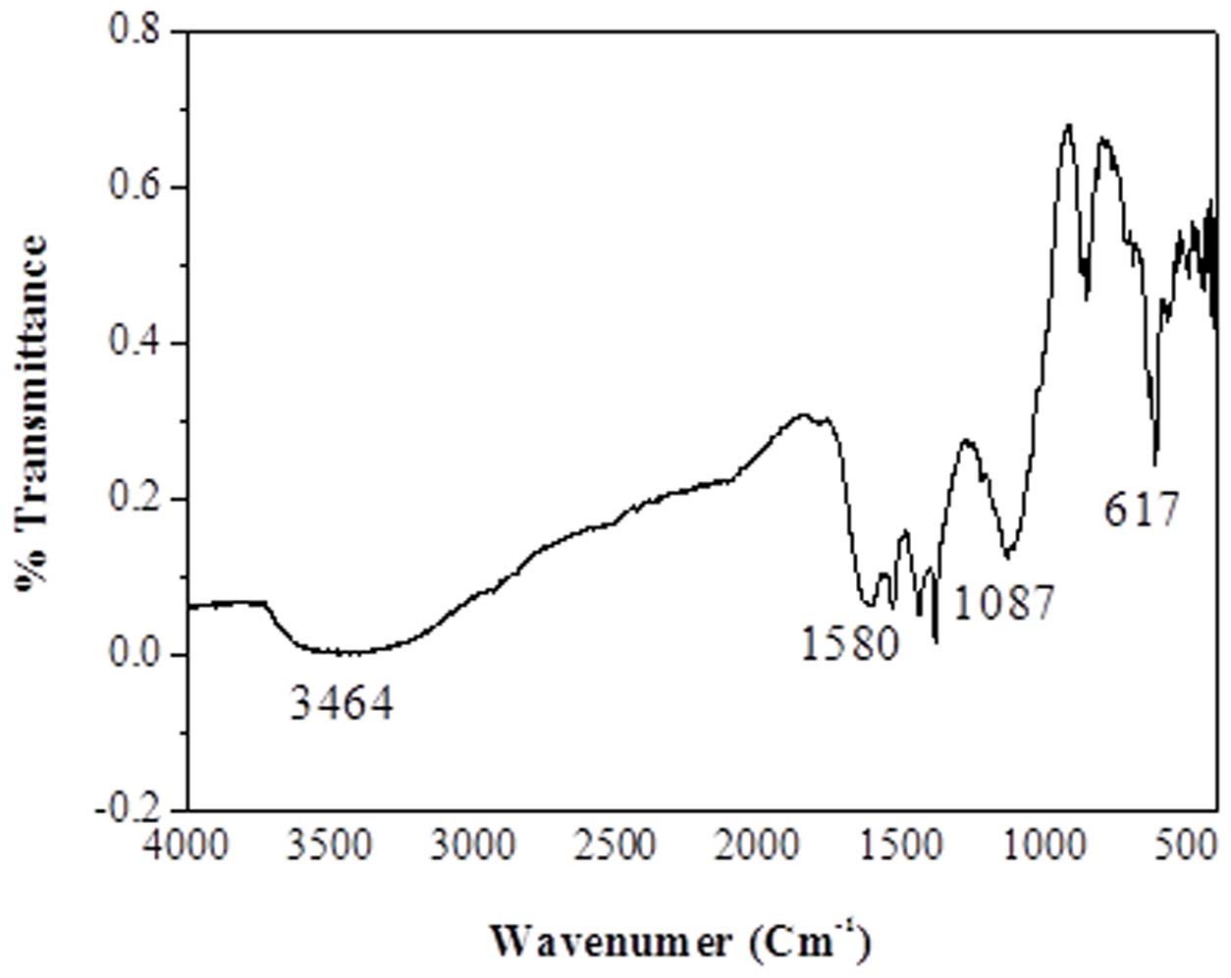
FT-IR spectrum of GCNC.

GCNC was prepared using copper acetate adsorbed graphene oxide (GO) sheets as precursors. In this composite, *in situ* formed Cu_2_O nanoparticles (NPs) were derived from the adsorbed copper acetate which attached to graphene sheets and prevented the aggregation of the reduced GO sheets. The synthesized Cu_2_O crystals were cube-like particles distributed randomly on the sheets due to the template effect of GO, consequently forming a GCNC. In fact, GO can also adsorb metal salts such as copper acetate on its surface. In this study, we have successfully synthesized GCNC with the aim to explore its structural and biological properties. The graphene oxide (GO) was synthesized employing the Hummers and Offeman [12] and collected by centrifugation. Under the specific conditions, the GO to GCNC was synthesized by the sodium borohydride reduction method. The synthesis reaction of GCNC indicated by gradual color change of the mixture, the initial dark yellow solution quickly turned dark brown and eventually became black within 10 min. We monitored the formation of GCNC by UV–vis absorption spectrum of GO shown in (Figure 1) is characterized by the *π*–*π*of the C = C plasmon peak around 230 nm and a shoulder around 300 nm which is often attributed to *n*–*π* transitions of the carbonyl groups. While GCNC (Figure 1b), the plasmon peak was red-shifts to ∼270 nm, reflecting increased *π*-electron concentration and structural ordering. Powder X-ray diffraction (XRD) analysis shown in (Figure 2a) demonstrates that the GCNC consists of cubic Cu_2_O (JCPDS 78-2076). The XRD data of GCNC indicates the absence of any impurities. The calculated average particle size was found to be ∼4 nm. Figure 2b shows the SEM microphotograph of GCNC. Graphene -Copper nanoparticles can be clearly seen in the image. Sample shows plate like forms without any other secondary phase. The results reveal that no obvious difference can be perceived in different parts of the sample, which further demonstrates that Cu contents are uniformly doped into the graphene matrix as demonstrated in the energy dispersive spectrum (EDS) as shown in Figure 2c. An oxygen peak at about 0.52 keV, Cu signals at about 1 keV, 8.0 keV and 9.0 keV and presence of the graphene at 0.25 keV were observed in the spectra. These results are consistent with the analysis of the XRD data. For further characterization TEM analysis was performed. The TEM image of GCNC is shown in (Figure 2d). At the low magnification, the structure of GCNC with wrinkles and folding on the surface was observed. Corrugation and scrolling are part of the intrinsic nature of graphene nanosheets, which results from the fact that the 2-D membrane structure becomes thermodynamically stable via bending. Figure 2d shows a high resolution TEM (HRTEM) image of GCNC. The crystal symmetry and well crystalline structure of the graphene nanosheets confirmed in the Figure 2d. To identify whether the GO sheets remain isolated or become aggregated, AFM was performed (Figure 3a and b). FT-IR spectrum of GCNC consists of the graphene and the Cu_2_O NPs. In the spectrum of GCNC, the strong absorption band at 617 cm^−1^ can be assigned to the vibrations of the Cu–O functional group (Figure 4).

**Figure 5:**
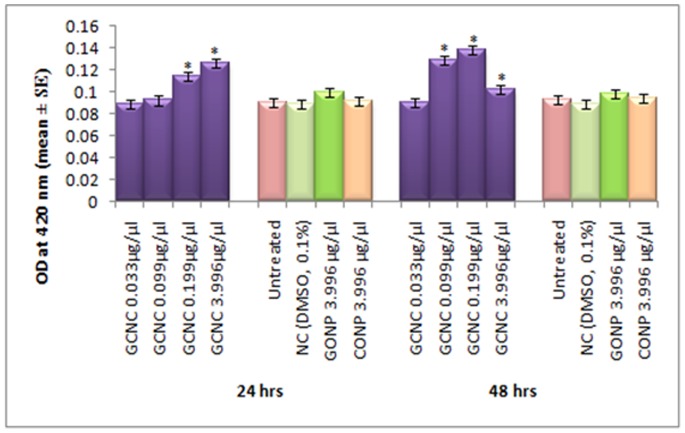
β-galactosidase activity measured in transgenic *Drosophila melanogaster(hsp70-lacZ)Bg^9^* third instar larvae exposed to different doses of Graphene copper nano composite (GCNC) for 24 and 48 hrs. *significant at p<0.05 with respect to untreated [GCNC = Graphene copper nano composite; GONP = Graphene oxide nano particle; CONP = Cuprous oxide nano particle; NC = Negative control; DMSO = Dimethyl sulphoxide; OD = Optical Density; SE = Standard error].

**Figure 6:**
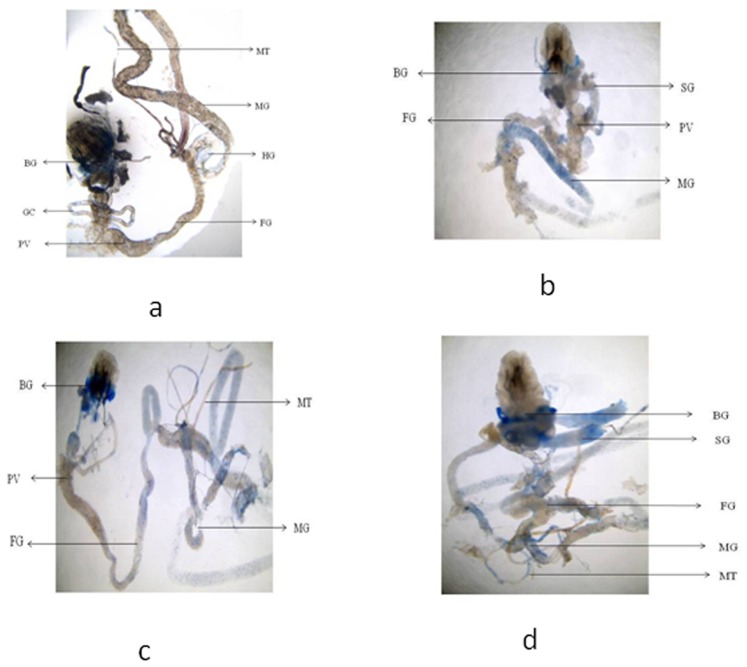
blue staining in the tissues of third instar larvae of transgenic *Drosophila melanogaster (hsp70-lacZ)Bg^9^* for untreated (a) and the larvae exposed to different doses of graphene copper nanocomposite (GCNC) for 48 hr of duration [0.099 µg/µl (b) 0.199 µg/µl (c) 3.996 µg/µl (d)]. [BG- Brain ganglia, SG- Salivary gland, PV- Proventriculus, FG- Foregut, MG-Midgut, HG- Hindgut, MT- Malpighian tubule, GC- Gastric caeca].

**Figure 7:**
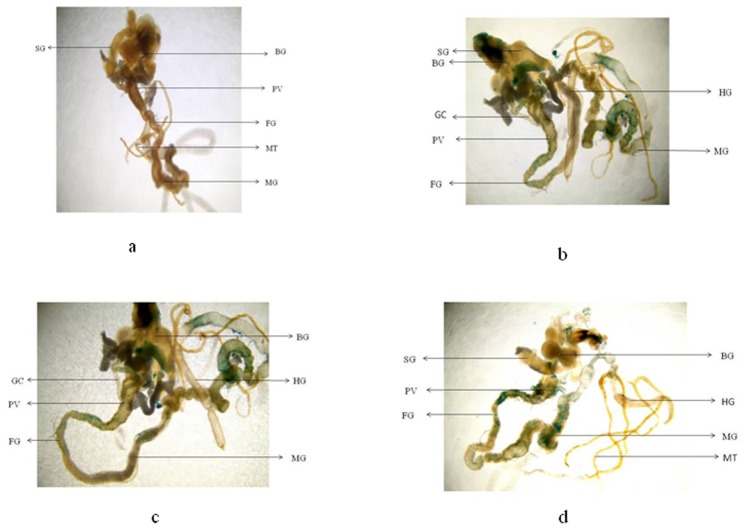
β-galactosidase staining pattern in the tissues of third instar larvae of transgenic *Drosophila melanogaster (hsp70-lacZ)Bg^9^* for untreated (a) and the larvae exposed to different doses of graphene copper nanocomposite (GCNC) for 48 hr of duration [0.099 µg/µl (b) 0.199 µg/µl (c) 3.996 µg/µl(d)]. [BG- Brain ganglia, SG- Salivary gland, PV- Proventriculus, FG- Foregut, MG-Midgut, HG- Hindgut, MT- Malpighian tubule, GC- Gastric caeca].

**Figure 8:**
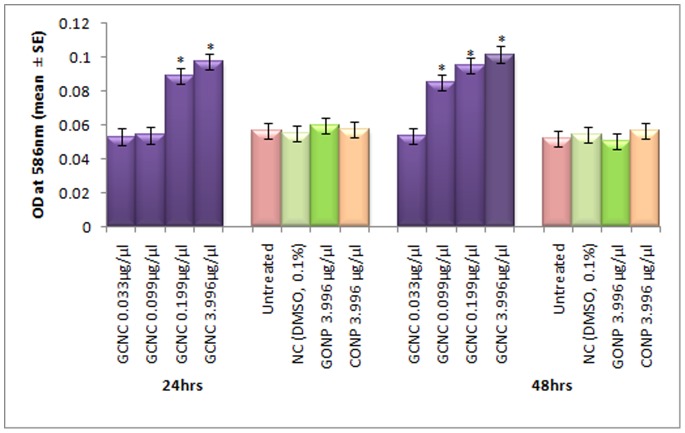
Lipid peroxidation in the third instar larvae of transgenic *Drosophila melanogaster (hsp70-lacZ)Bg^9^* exposed to different doses of Graphene copper nano composite (GCNC) for 24 and 48 hrs. *significant at p<0.05 with respect to untreated[GCNC = Graphene copper nano composite; GONP = Graphene oxide nano particle; CONP = Cuprous oxide nano particle; NC = Negative control; DMSO = Dimethyl sulphoxide; OD = Optical Density; SE = Standard error].

**Figure 9:**
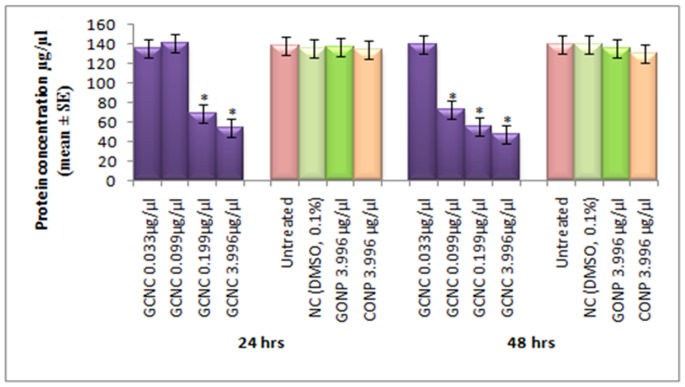
Protein content in the third instar larvae of transgenic *Drosophila melanogaster(hsp70-lacZ)Bg^9^* exposed to different doses of Graphene copper nano composite (GCNC) for 24 and 48 hrs. *significant at p<0.05 with respect to untreated [GCNC = Graphene copper nano composite; GONP = Graphene oxide nano particle; CONP = Cuprous oxide nano particle; NC = Negative control; DMSO = Dimethyl sulphoxide; SE = Standard error].

**Figure 10:**
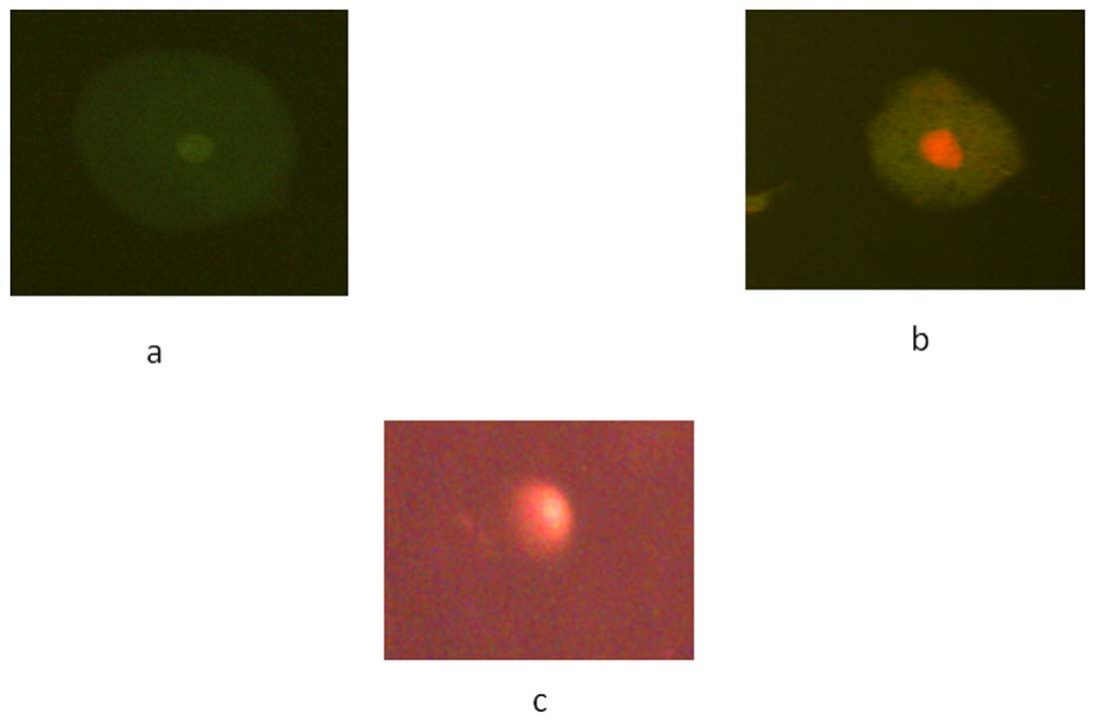
*Drosophila melanogaster(hsp70-lacZ)Bg^9^* mid gut cells (a) Normal cell; (b) Apoptotic cell and (c) Comet assay performed in gut cell exposed to 3.996 µg/µl of GCNC for 48 hrs of duration.

**Figure 11:**
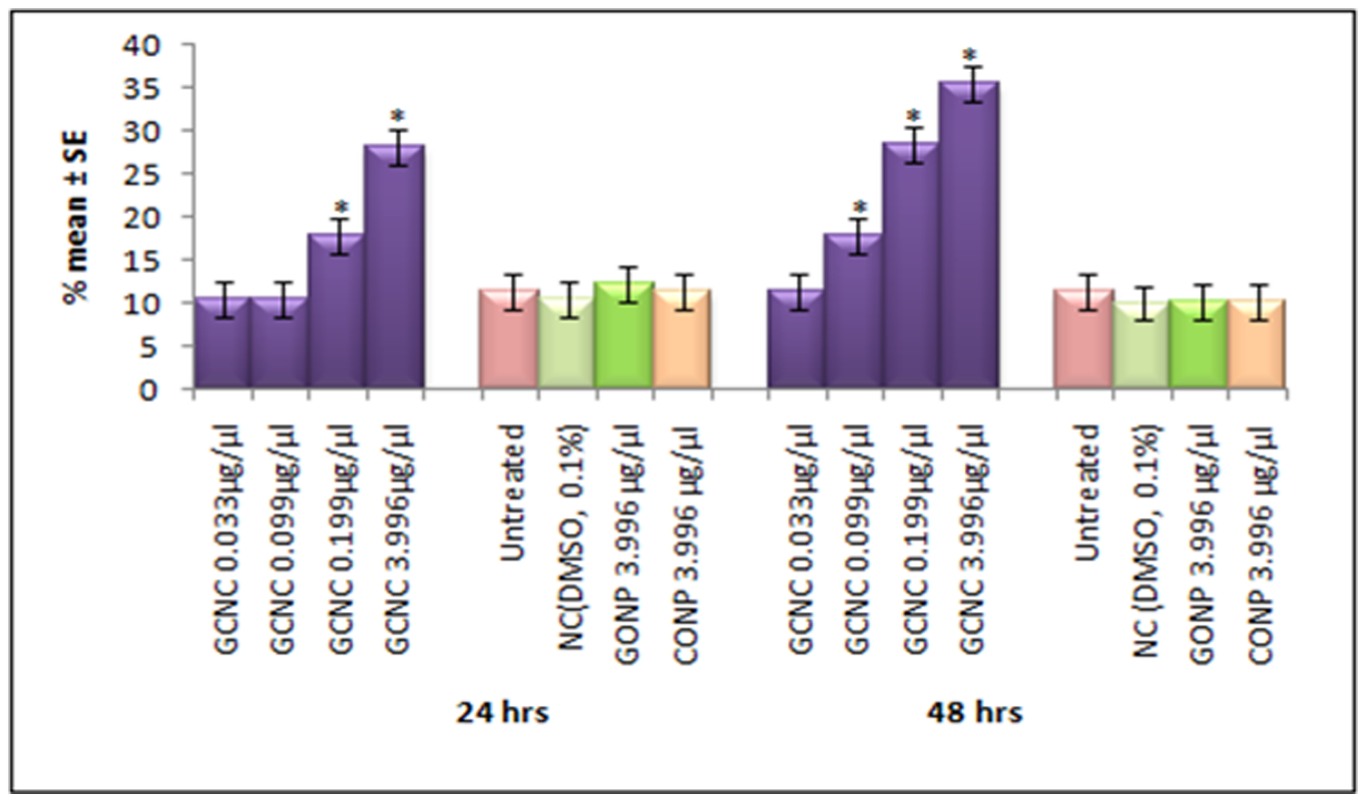
Apoptotic index measure in the midgut cells of the third instar larvae of transgenic *Drosophila melanogaster(hsp70-lacZ)Bg^9^* exposed to different doses of graphene copper nano composite (GCNC) for 24 and 48 hrs. *significant at p<0.05 with respect to untreated [GCNC = Graphene copper nano composite; GONP = Graphene oxide nano particle; CONP = Cuprous oxide nano particle; NC = Negative control; DMSO = Dimethyl sulphoxide; SE = Standard error].

**Figure 12:**
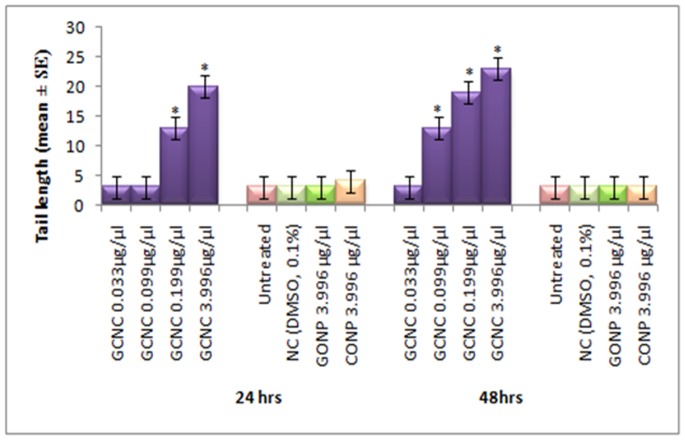
Comet assay performed on the midgut cells of the third instar larvae of transgenic Drosophila melanogaster(hsp70-lacZ)Bg9 exposed to different doses of Graphene copper nano composite (GCNC) for 24 and 48 hrs. *significant at p<0.05 with respect to untreated [GCNC = Graphene copper nano composite; GONP = Graphene oxide nano particle; CONP = Cuprous oxide nano particle; NC = Negative control; DMSO = Dimethyl sulphoxide; SE = Standard error].

The expression of *hsp70* in the third instar larvae of transgenic *Drosophila melanogaster (hsp70-lacZ)Bg^9^* was measured both quantitatively and qualitatively. Larvae exposed to GCNC at 0.033 and 0.099 µg/µl for 24 hr did not show significant increase in the expression of β-galactosidase (Figure 5). However, the larvae exposed to 0.199 and 3.996 µg/µl for 24 hr showed a dose dependent significant increase in the expression of β-galactosidase as compared to untreated (Figure 5). For 48 hr of exposure to a dose at 0.033 µg/µl did not show significant increase in the expression of β-galactosidase as compared to untreated (Figure 5). GONP (3.996 µg/µl) and CONP(3.996 µg/µl) did not show significant increase in the expression of β-galactosidase as compared to untreated for both 24 as well as 48 hrs of exposure (Figure 5). The exposure of larvae to 0.099, 0.199 and 3.996 µg/µl for 48 hr showed a dose dependent significant increase in the expression of β-galactosidase compared to untreated (Figure 5). Figure 6 (b–d) shows the trypan blue staining in the third instar larvae exposed to various doses of GCNC for 48 hr of duration. About 95% of untreated larvae were negative to the trypan blue staining (Figure 6a). The exposure of larvae to 0.033 µg/µl of GCNC did not show any tissue damage, but the exposure of larvae to 0.199 and 3.996 µg/µl of GCNC show a dose dependent tissue damage for 24 as well as 48 hrs of exposure. A dose dependent tissue damage was observed in salivary glands, foregut, midgut and malphighian tubules. The larvae exposed to GONP (3.996 µg/µl) and CONP (3.996 µg/µl) for 24 as well as 48 hrs were negative to trypan blue staining (Figures not shown). Figure 7 (a–d) shows β-galactosidase staining in untreated and the larvae exposed to 0.099, 0.199 and 3.996 µg/µl of GCNC for 48 hr. Dose dependent moderate to dark blue staining in the foregut, midgut and malphighian tubules was observed. The same results were observed for 24 hr of exposure to 0.099 and 0.199 µg/µl of GCNC (figures not shown). The exposure of larvae to GONP (3.996 µg/µl) and CONP (3.996 µg/µl) for 24 as well 48 hrs were negative to β-galactosidase staining (Figures not shown). The results obtained for lipid peroxidation are shown in Figure 8. The treatment of 0.033 and 0.099 µg/µl of GCNC for 24 hr did not show any significant increase in mean absorbance values for the estimation of lipid peroxidation (Figure 8). The exposure of 0.199 and 3.996 µg/µl of GCNC for 24 hr of duration showed a significant increase in the mean absorbance values i.e 0.0890±0.0008 and 0.0976±0.0005, respectively, as compared to untreated (Figure 8). The exposure of 0.033 µg/µl of GCNC for 48 hr did not show any significant increase in the mean absorbance values as compared to untreated, but the exposure of 0.099, 0.199 and 3.996 µg/µl of GCNC for 48 hr to the third instar larvae of transgenic *Drosophila melanogaster (hsp70-lacZ)Bg^9^* showed a dose dependent significant increase in the mean absorbance value i.e. 0.0852±0.0013, 0.0954±0.0007 and 0.1016±0.0007, respectively as compared to untreated (Figure 8).). The exposure of larvae to GONP (3.996 µg/µl) and CONP (3.996 µg/µl) for 24 as well 48 hrs did not show significant increase in the mean absorbance values as compared to untreated (Figure 8). The effect of GCNC on total protein content is shown in Figure 9. No significant difference in total protein content was found as compared to untreated in the third instar larvae exposed to 0.033 and 0.099 µg/µl of GCNC for 24 hr but a significant decrease in the protein content as compared to untreated was observed at 0.199 and 3.996 µg/µl (Fig. 9). The larvae exposed to 0.033 µg/µl of GCNG for 48 hr showed no significant decrease in the protein content but at the doses of 0.099, 0.199 and 3.996, a significant dose dependent decrease in the total protein content was observed i.e. 72.50±1.456, 55.83±0.948 and 46.76±0.830, respectively (Figure 9).). The larvae exposed to GONP (3.996 µg/µl) and CONP (3.996 µg/µl) for 24 as well 48 hrs did not show significant difference in the total protein content as compared to untreated (Figure 9). The normal and apoptotic midgut cells of the third instar larvae are shown in Figure 10. Apoptotic index for the midgut cells of the third instar larvae exposed to different doses of GCNC for 24 and 48 hrs of duration is shown in Figure 11. The exposure of larvae to 0.033 and 0.099 µg/µl for 24 hr did not show any significant increase in the apoptotic index as compared to the untreated (Figure 11). The exposure of 0.199 and 3.996 µg/µl of GCNC for 24 hr showed a significant increase in the apoptotic index as compared to the untreated (Figure 11). The exposure of 0.033 µg/µl of GCNC for 48 hr did not show any significant increase in the apoptotic index as compared to untreated, but the exposure of 0.099, 0.199 and 3.996 µg/µl of GCNC showed a dose dependent increase in the value of apoptotic index i.e 17.80±0.860, 28.40±0.400 and 35.40±0.678, respectively (Figure 11).). The exposure of larvae to GONP (3.996 µg/µl) and CONP (3.996 µg/µl) for 24 as well 48 hrs did not show significant increase in the apoptotic index as compared to untreated (Figure 11). Comet assay performed on the midgut cells of the third instar larvae of transgenic *Drosophila melanogaster (hsp70-lacZ)Bg^9^* is shown in Figure 10. The result obtained for the comet assay performed for the midgut cells of the third instar larvae is shown in Figure 9. The exposure of larvae to 0.033 and 0.099 µg/µl of GCNC for 24 hr did not show any significant increase in the mean tail length (Figure 12). The exposure of 0.199 and 3.996 µg/µl of GCNC showed a significant increase in the mean tail length i.e. 13±0.583 and 20±1.00, respectively, as compared to untreated (Figure 12). The exposure of larvae to 0.033 µg/µl of GCNC for 48 hr of duration did not show any significant increase in the mean tail length (Figure 12). The exposure of 0.099, 0.199 and 3.996 µg/µl of GCNC for 48 hr of duration showed a dose dependent significant increase in the mean tail length i.e 13±0.0583, 19±0.510 and 23±0.316, respectively, as compared to untreated (Fig. 12). The exposure of larvae to GONP (3.996 µg/µl) and CONP (3.996 µg/µl) for 24 as well 48 hrs did not show any increase in the mean tail length as compared to untreated (Figure 12). The results obtained for the synthesis of GCNC i.e. the color change was indicative of the formation of suspended GCNC that appeared black and insoluble [23]. The results of the absorption spectrum are consistent with the restoration of sp^2^ carbon and possible rearrangement of atoms [24]. It implies that the GO might be reduced and the aromatic structure might be restored gradually. Similar features and trends are observed for the reduction of GO by L-ascorbic acid [25]. The results obtained for the X-ray diffraction (XRD) shows no graphite diffraction peaks are present in the XRD pattern, indicating that the regular stack of GO has been broken [26]. The morphology of graphene as circled in the image is observed as a flaky texture reflecting its layered microstructure and Cu particles are dispersed on the surface of graphene or imbedded into the graphene sheets [27], [28]. Furthermore it is clearly observed that the graphene nanosheets are covered by densely packed and irregularly shaped Cu grains, spreading in a large-scale. The stretching vibrations of carboxyl groups or conjugated carbonyl groups cannot be observed, which means the reduction of graphene to GO is completed [29]. The absorption band at 1580 cm^−1^ can be assigned to the stretching vibration of C = C of graphene and another at 1087 cm^−1^ can be assigned to the stretching vibration of C–O of graphene [30].

The results of the present study show that higher doses GCNC are toxic as is evident from the results obtained in our study. Graphene has attracted tremendous interest in different areas including biomedicine. Multiple drugs therapy is widely use in cancer therapy [10]. As nano carriers GO co-loaded with two chemical drugs, doxorubicin and captothecin was studied on MCF-7 cells, the higher cytotoxicity was observed in the MCF-7 cells [11]. GO nanocomposites using different drugs have been studied on various cell lines to explore GO-based drug delivery [31], [32]. The carboxyl functionalization of graphene in pacifying its strong hydrophobic interaction with cells is associated with the toxic effects [33]. GO has been reported to be deposited predominantly in the lungs. No pathological changes were observed in the organs of mice at 1 mg/kg body weight of GO for 14 days, but at 10 mg/kg body weight pulmonary edema and granuloma were observed [34]. A dose dependent toxicity in human fibroblast cell of GO at 50 µg/µl has been reported by Wang et al [35]. The dietary uptake of fullerene C60, carbon black (CB), or single walled or multi walled nanotubes (SWNTs, MWNTs) were studied on larval stage, and adults of *Drosophila melanogaster*. No detectable effects on egg to adult survivorship, despite the evidence of uptake were observed [36]. However, the exposure of the same materials in dry form to adult *D. melanogaster*, some materials (CB and SWNTs) led to impaired locomotor function and mortality [36]. In the present study the β-galactosidase acitivity was used as an indicator of expression of *Hsp70*. β-galactosidase activity was also confirmed by X-gal staining for the third instar larvae. Stress inducible *Hsp70* has been reported as a first tier bioindicator of cellular damage due to its conservation through evolution, inducibility by a wide variety of inducers and being a part of the cellular defense machinery [37], [38]. Tissue damage evaluated by trypan blue staining showed more damage at the highest concentration for the exposure of 48 hr which is correlated by the reduced activity of β-galactosidase at 3.996 µg/µl. The slight reduction in the expression of β-galactosidase for the exposure of third instar larvae at 3.996 µg/µl for 48 hr may be due to the damage in the tissues at this dose and reduction in the number of viable cells due to auto-repression of *Hsp70* once its upper limit has been achieved. The instability of the reporter gene may also be involved for the exposure at this dose that may results in the decrease expression of *Hsp70.* A dose dependent decrease in the total protein content is clearly correlated (Table 1) with the increased lipid peroxidation [r = −0.9978, p<0.0008 (24 hr); r = −0.998, p<0.0003(48 hr)] and apoptotic index [r = −0.9216, p<0.255 (24 hr); r = −0.8850, p<0.0302 (48 hr)]. It has been suggested that proteins are the targets for the oxidants as a result of their abundance in biological systems and can be used as an indicator of the cytotoxicity [39], [40]. Copper oxide nano particle have been reported not only to generate the reactive oxygen species (ROS), but also to block the cellular antioxidant defenses [41]. Thus, higher protein damage in the exposed larvae caused by ROS may be one of the possible reasons of apoptosis. ROS have been suggested to play a major role in enhancing the toxicity of several xenobiotics including nano particles [34]. Although the cells are well acquaint with the self defense mechanisms, but an enhancement in the stress beyond the capacity of a cell to cope up may result in the cellular damage leading to the cell death [42]. The resulting ROS can damage lipid, protein and DNA [43]. Lipid peroxidation is considered a reliable marker of oxidative stress [44]. GCNC induced DNA damage was observed in *Drosophila* larval midgut cells as evidenced by a significant increase in the mean tail length in the comet assay performed for the exposed larvae at higher doses. Along with the DNA damage increase in the apoptotic index was observed. A positive correlation (Table 1) was observed in DNA damage and apoptotic index [(r = 0.9844, p<0.0450 (24 hr); 0.9690, p<0.2326 (48 hr)]. A negative correlation observed between the β-galactosidase expression and protein level clearly demonstrates the proteotoxicity in the larvae exposed to higher doses of GCNC, for 24 and 48 hr of duration i.e [r = −0.9815; p<0.0100 and r = −0.6140; p<0.223, respectively (Table 1).

**Table 1:** Regression analysis for *hsp70* expression, lipid peroxidation, protein content, apoptosis and comet tail length in the third instar larvae of transgenic *Drosophila melanogaster (hsp70-lacZ)Bg^9^*.

		24 hr				48 hr			
S.No.	Groups	Regression equation	r	p	F	Regression equation	R	p	F
1.	β_gal_ vs L	Y_L_ = −0.0629 + 1.2962X_gal_	0.99022	<0.044	100.7134	Y_L_ = 0.02201 + 0.53814 X_gal_	0.57200	<0.7625	0.9725641
2.	β_gal_vs Ap	Y_Ap_ = −31.42 + 457.61X_gal_	0.96493	<0.0784	27.02286	Y_Ap_ = 9.0767 + 123.25 X_gal_	0.26204	<0.8311	0.1474499
3.	β_gal_vs CTL	Y_CTL_ = −39.09 + 464.47 X_gal_	0.99371	<0.0100	157.4825	Y_CTL_ = −5.363 + 172.72X_ gal_	0.45225	<0.8663	0.5142355
4.	β_gal_ vs P	Y_P_ = 359.11 − 2466 X_gal_	−0.9815	<0.0100	52.64479	Y_P_ = 208.32 − 1128 X_gal_	−0.6140	<0.2238	1.210328
5.	L vs Ap	Y_Ap_ = −8.024 + 336.84 X_L_	0.92975	<0.3799	12.75209	Y_Ap_ = −15.04 + 456.63X_L_	0.91284	<0.3491	9.996292
6.	L vs CTL	Y _CTL_ = −15.93 + 349.92 X_ L_	0.97995	<0.0532	48.38880	Y_CTL_ = −19.06 + 400.01 X_L_	0.98536	<0.0454	66.82125
7.	Lvs P	Y_P_ = 240.40 − 1915 X_L_	−0.9978	<0.0008	454.8722	Y_P_ = 242.10 − 1949X_L_	−0.9980	<0.0003	487.5854
8.	Ap vs P	Y_P_ = 181.38 − 4.882 X_Ap_	−0.9216	<0.255	11.28187	Y_P_ = 158.96 + 3.457X_Ap_	−0.8850	<0.0302	7.223134
9.	Ap vs CTL	Y_CTL_ = −6.453 + 0.9702 X_Ap_	0.98440	<0.0450	62.59781	Y_CTL_ = −3.795 + 0.7868 X_Ap_	0.96905	<0.2326	30.81874
10.	P vs CTL	Y_CTL_ = 27.866–0.1814 X_P_	−0.9751	<0.0046	38.60752	Y_CTL_ = 30.389 − 0.2022 X_P_	−0.9725	<0.0077	34.82287

β_gal_ = β-galactosidase, L = Lipid peroxidation, Ap = Apoptosis, P = Protein, CTL = Comet tail length.

*Drosophila* as a model organisms have been used to study the toxic effects of nano particles. The CdSe-ZnS quantum dots affected the life span of *Drosophila* and also increase the levels of reactive oxygen species (ROS). [45]. The genotoxicity of cobalt nano particles (CoNPs) and ions were evaluated using *Drosophila* as a model. CoNPs as well as the ionic cobalt chloride at concentration ranging from 0.1 to 10 mM were able to induce significant increase in the frequency of mutant clones [46]. In other study with copper oxide nanoparticles on human lung epithelial cells, the CUONPs reduced the cell viability, deplete glutathione, induce lipid peroxidation, catalase and superoxide dismutase and increase the *Hsp70* expression in dose dependent manner [47]. The genotoxic potential of CUONPs was attributed to the oxidative stress [47]. The first nanomaterial mutated organism, named MN-mut (*Drosophila melanogaster*) was obtained by exposing the flies to gold nanoparticles (AUNPs). The modified phenotypes in subsequent generations were observed suggesting the capability of AUNPs to induce mutagenic effects that may be transmitted to the descendants [48]. Metals have been reported as inducers of *Hsp70* [49]. Copper produces free radicals, and when present in an unbound condition, it produces reactive oxygen species that cause DNA, protein and lipid damage [50]. Midgut of insects has been reported to be rich in cytochrome –P450 species [51]. Hence the midgut cells were taken for the comet assay and apoptotic index analysis. The method used in the present study for the apoptosis analysis is simple as there is no cell fixation step thus avoiding a number of potential artifacts [52]. Other methods of detecting the apoptosis involves multiple steps (Annexin V, DNA ladder), lack of ability to quantify percent of live, apoptotic and necrotic cells at the same time (DAPI staining, Caspase 3/7 activity, DNA laddering and SS DNA staining), and are non specific (TUNEL assay [21]. These methods may damage the cell membranes and change the cell population, distribution of live, apoptotic and necrotic cells. The blood compatibility and cytotoxicity of graphene oxide as graphene sheets of various sizes and oxygen content was investigated in suspended human RBCs and adherent skin fibroblasts using *in vitro* haemolysis and WST-8 viability assays. All the tested doses of GO and GS showed the dose dependent haemolytic activity and toxicity to the adherent skin fibroblasts [6]. In another study of graphene oxide, carried out on A549 cells and PC12 cells, a dose dependent cytotoxicity has been attributed to ROS [1], [25]. Nanomaterials have unique physicochemical properties and are applied in various areas. However, their biological properties in organisms will finally determine their destiny in future [1]. In our present study with GCNC was toxic only at higher doses and longer duration of exposure. The dose 0.033 µg/µl may be considered as the No Observed Adverse Effect Level (NOAEL). Due to ethical reasons, exorbitant cost and difficulty in interpreting data owing to interspecies variation, the issues related to the use of animals in toxicology research and testing have become serious concerns among scientists [53]. This has led to the scientist to promote the use of an alternative to higher animals in toxicology. A *Drosophila* is a well established model for pharmacological or toxicological evaluations [54]. A systematic and reproducible evaluation of nanoparticles (NPs) toxicology in living systems using the ingestion of citrate-capped gold NPs (AUNPs) of different sizes by the model system *Drosophila melanogaster* has been well documented and have pave the way for the risk assessment and regulatory approval for various nanoparticles and nanomedicine applications [55].

### Conclusions

In conclusion, the toxicity of GCNC was observed in the third larvae of transgenic *Drosophila melanogaster (hsp70-lacZ)Bg^9^*. Hence the full implementation of such nano materials as biological applications needs to be more investigated.
