# Correction: Evaluation of the Toxic Potential of Graphene Copper Nanocomposite (GCNC) in the Third Instar Larvae of Transgenic *Drosophila melanogaster (hsp70-lacZ)Bg^9^*

**DOI:** 10.1371/journal.pone.0093127

**Published:** 2014-03-26

**Authors:** The PLOS ONE Staff

There was an error in the first sentence of the second paragraph of the “Characterization of GCNC” sub-heading of the Materials and Methods section. The correct sentence is: The average crystallite size (D) of Cu2O NPs was calculated following the Debye-Scherrer formula.

There was an error in the first sentence of the last paragraph of the “Characterization of GCNC” sub-heading of the Materials and Methods section. The correct sentence is: Where k  =  0.9 is the shape factor, λ is the X-ray wavelength of Cu Kα radiation (1.54 Å), θ is the Bragg diffraction angle, and β is the full width at half maximum height (FWHM) of the (III) plane diffraction peak.

There was an error in the third sentence of the last paragraph of the “Characterization of GCNC” sub-heading of the Materials and Methods section. The correct sentence is: For the morphological analysis transmission electron microscopy (TEM) of ethanol solution of GCNC was carried out on JEOL 100/120 kV transmission electron microscope (JEOL, Tokyo, Japan) with an accelerating voltage of 200 kV.

There was an error in the fifth sentence the last paragraph of the “Characterization of GCNC” sub-heading of the Materials and Methods section. The correct sentence should read: For SEM measurement the thin film of the GCNC was prepared on the borosilicate glass slide for the analysis of surface morphology.

The fifth to last sentence of the first paragraph of the Results and Discussion section, “Figure 2d shows a high resolution TEM (HRTEM) image of GCNC,” should be deleted.
